# Soluble and bacteriophage T4 displayed gp41 mutant proteins as HIV-1 vaccine candidates

**DOI:** 10.1186/1742-4690-9-S2-P10

**Published:** 2012-09-13

**Authors:** G Gao, KK Peachman, L Wieczorek, V Polonis, CR Alving, M Rao, VB Rao

**Affiliations:** 1 The Catholic University of America, Washington, DC, USA; 2 USMHRP, Walter Reed Army Institute of Research, Silver Spring, MD, USA

## Background

HIV-1 envelope protein gp41 is a very attractive vaccine target as its epitopes are recognized by three broadly neutralizing antibodies. However, the extreme hydrophobicity and very transient exposure of neutralizing epitopes during infection has hampered its usage in HIV-1 vaccine development. Our goal is to design a soluble trimeric gp41 vaccine stabilized as a pre-hairpin intermediate.

## Methods

A gp41AVERY-minus recombinant was constructed, which contained both gp41 ecto-domain and cyto-domain, while the immunodominant AVERY region was deleted to reduce elicitation of non-neutralizing antibodies. Another protein, gp41-5M, was constructed by introducing 5 point mutations to gp41AVERY-minus, at the exposed hydrophobic residues of the trimeric coiled coil region, in order to destabilize the interaction between HR1 and HR2 helices and therefore mimic the gp41 pre-hairpin intermediate. A ‘foldon’ structural tag was fused at the C-terminus of gp41-5M to facilitate trimer formation, and phage T4 small outer capsid (Soc) protein was fused at the N-terminus for arraying gp41 on T4 capsids. The ability of purified Soc-gp41-5M proteins to inhibit 4E10 and 2F5 neutralizing activity was tested in the TZM-bl assay. Immunization experiments were performed in rabbits using soluble as well as T4 displayed gp41 antigens to determine their immunogenicity.

## Results

gp41 mutant proteins were over-expressed in E. coli and purified in soluble form following denaturation, renaturation, and Histrap affinity chromatography. The Soc-gp41-5M protein formed trimers and other oligomers, and effectively competed with native epitopes present on HIV-1 virus for binding to 4E10 and 2F5 antibodies in the TZM-bl assay. The sera from all immunized rabbits showed binding antibodies to gp41 while some also showed neutralizing antibodies.

## Conclusion

Soluble near full-length trimeric gp41 immunogens can be produced by interfering with the HR1-HR2 interactions while retaining the 4E10 and 2F5 binding epitope conformations. These proteins might be good vaccine candidates to elicit broadly neutralizing antibodies.

